# Ethyl acetate extract of *Elephantopus mollis* Kunth induces apoptosis in human gastric cancer cells

**DOI:** 10.1186/s12906-021-03444-6

**Published:** 2021-10-30

**Authors:** Tran Dang Thanh Tam, Truong Thi Bich Ngoc, Nguyen Thi Hoai Nga, Nguyen Thi My Trinh, Tran Linh Thuoc, Dang Thi Phuong Thao

**Affiliations:** 1 Department of Molecular and Environmental Biotechnology, Faculty of Biology and Biotechnology, VNU-HCM, University of Science, 227 Nguyen Van Cu, Ho Chi Minh City, 700000 Vietnam; 2 Laboratory of Molecular Biotechnology, VNU-HCM, University of Science, 227 Nguyen Van Cu, Ho Chi Minh City, 700000 Vietnam; 3 Laboratory of Cancer Research, VNU-HCM, University of Science, Duong so 4, Linh Trung, Thu Duc, Ho Chi Minh City, 700000 Vietnam; 4 Vietnam National University, Ho Chi Minh City, Vo Truong Toan, Linh Trung, Thu Duc, Ho Chi Minh City, 700000 Vietnam

**Keywords:** *Elephantopus mollis* Kunth, Ethyl acetate extract, Gastric cancer, Cytotoxicity, Apoptosis

## Abstract

**Background:**

Gastric cancer is one of the most leading causes of cancer death worldwide. Therefore, treatment studies have been being conducted, one of which is screening of novel agents from medicinal herbs. *Elephantopus mollis* Kunth (EM) belonging to *Asteraceae* family is a perennial herb with several therapeutic properties including anticancer activity. However, the effect of this species on gastric cancer has not been reported yet. In this study, cytotoxicity of different EM crude extracts was investigated on AGS gastric cancer cell line. Besides, the effects of extract on nuclear morphology, caspase-3 activation, and gene expression were also explored.

**Results:**

The results showed that ethyl acetate extract exhibited a remarkably inhibitory ability (IC_50_ = 27.5 μg/ml) on the growth of AGS cells, while causing less toxicity to normal human fibroblasts. The extract also induced apoptotic deaths in AGS cells as evidenced by cell shrinkage, formation of apoptotic bodies, nuclear fragmentation, caspase-3 activation, and the upregulation of *BAK* and *APAF-1* pro-apoptotic genes related to mitochondrial signaling pathway. Specifically, *BAK* and *APAF-1* mRNA expression levels showed 2.57 and 2.71-fold increases respectively.

**Conclusions:**

The current study not only proved the anti-gastric cancer activity of EM ethyl acetate extract but also proposed its molecular mechanism. The extract could be a potential candidate for further investigation.

## Background

Gastric cancer is the fifth most frequently diagnosed cancer and third leading cause of cancer death worldwide, with over 1 million newly diagnosed cases and 783,000 deaths each year [1]. Although there have been efforts in *Helicobacter pylori* infection treatment [2, 3] and earlier diagnosis [4], the mortality incidence remains high [1]. For gastric cancer, surgery is the main curative therapy, while chemotherapy or chemoradiation can be used in combination [5]. However, the relapse of tumor [6], drug resistance [7], metastasis of cancer cells [8], and side effects of chemotherapy [9, 10] present as challenges affecting treatment efficacy and shortening survival time of patients. For those reasons, studies on gastric cancer treatment are ongoing, among which is screening of novel anticancer agents from nature [11]. Medicinal herbs are popularly used in folk medicine with several therapeutic properties, so they are considered as potential sources for investigation.

*Elephantopus mollis* Kunth (EM) belonging to *Asteraceae* family is a perennial herb up to 40 cm tall. This species is native to Central and South America, but also appears in Africa, Eastern Asia, Australia and Pacific Islands. In traditional folk medicine, EM is used as a febrifuge, diuretic, and antidote for snakebite. The whole plant is utilized to treat diarrhea, while leaves are used for ulcers and eczema [12]. In scientific reports, EM has been proved antibacterial [13], anti-inflammatory [14], and anticancer activities. The crude extracts of EM could inhibit varied human cancer cell lines, including breast carcinoma [15, 16], lung carcinoma [15–17], liver carcinoma [15, 16], leukemia [17], and colorectal carcinoma [15]. Regarding molecular mechanism, ethyl acetate extract could induce apoptotic deaths in HepG2 liver carcinoma cells as indicated by DNA fragmentation and caspase-3 activation [16], while methanol extract caused loss of mitochondrial integrity and exerted the increase in reactive oxygen species (ROS) level in MCF-7 breast carcinoma cells [16]. The ethyl acetate extract could also trigger apoptosis in A549 lung carcinoma cells by upregulating pro-apoptotic genes (*BAK* and *BID*) and downregulating anti-apoptotic gene *BCL-2* [17]. Some triterpenes, sesquiterpene lactones and phenolic acid compounds from this species exhibited substantial anticancer activity [18–21]. Specifically, EM23 compound, a sesquiterpene lactone, was toxic to chronic and acute leukemia cells (IC_50_ = 10.8 and 1.9 μM respectively) due to thioredoxin system inhibition leading to the increase in intracellular ROS level, the activation of ASK-1/MAPK pathway and apoptotic deaths [21]. In addition, recently, EM2, a monomeric sesquiterpene lactone called 2β-methoxy-2- deethoxyphantomolin, was proved to inhibit liver carcinoma cells (IC_50_ = 5.89 μM) by causing G2/M arrest, exerting endoplasmic reticulum stress, and activating JNK pathway [22]. Thus, it is obvious that although anticancer activity of EM has been studied since 1970s, its potential has not been fully exploited yet.

Although there are several studies on anticancer activity of EM, no data has been recorded on its capacity towards gastric cancer yet. Therefore, in our study, cytotoxicity of EM crude extracts was investigated on AGS human gastric adenocarcinoma cell line.

## Methods

### Plant materials

*Elephantopus mollis* Kunth (local name: “cúc chỉ thiên mềm”, English name: soft elephant’s foot) was harvested in Bidoup - Nui Ba Vietnam National Park, Lam Dong Province, Vietnam. The plant was identified by Laboratory of Plants, University of Science, Vietnam National University, Ho Chi Minh City, Vietnam (voucher specimen: PHH0004880).

### Chemicals

Solvents including ethanol, ethyl acetate and petroleum ether were purchased from Xilong (China). DMSO (99.9% (v/v)) was supplied by Sigma-Aldrich (Germany). Cell culture media consisting of RPMI-1640 and high glucose DMEM were obtained from Himedia (India) and Sigma-Aldrich (Germany). Fetal bovine serum (FBS), trypsin and MTT (3-(4,5-Dimethylthiazol-2-yl)-2,5-diphenyltetrazolium bromide) were supplied by Sigma-Aldrich (Germany). Doxorubicin was supplied by Ebewe Pharma (Austria). Paraformaldehyde and Triton-X100 were obtained from Scharlau (Spain) and Pancreac AppliChem (Spain-Germany-Italia). Anti-cleaved caspase-3 antibodies (#9661), secondary antibodies (#A-11008), and normal goat serum (NGS) were purchased from Cell Signaling Technology (USA), Invitrogen (USA), and Vector Laboratories (Japan) respectively. TriSure Kit for RNA extraction, Tetro cDNA Synthesis Kit, and SensiFAST™ HRM Realtime PCR Kit were obtained from Bioline (UK). qPCR primers were supplied by PhuSa (Vietnam).

### Plant extract preparation

Dried whole-plant materials were ground into powder. Next, the plant powder was immersed in 70% ethanol (ratio 1:10 (w/v)). Then, the whole solution was collected, filtered and concentrated by a rotary evaporator (Hahnvapor, Korea). After that, part of the solution was dried by a freeze drier (Benchtop K Virtis, USA) to obtain ethanol-extract powder. The remaining solution was sequentially extracted with petroleum ether and ethyl acetate solvents (ratio 1:1 (v/v)), then also concentrated by the rotary evaporator and dried by the freeze drier to gain powders of petroleum ether, ethyl acetate and water extracts. The efficiency of total extraction was 8.7% (w/w) based on the dry weight of whole-plant materials.

The extract powders were dissolved in 99.9% (v/v) DMSO (Merck, Germany) and stored at -20 °C. Before experiments, the extracts were diluted in PBS solution, then filtered by sterile 0.22-μm membranes.

### Cell line culture

Two cell lines were used in this study, including AGS human gastric adenocarcinoma (ATCC, CRL-1739) and BJ-5ta human normal fibroblast immortalized with hTERT (ATCC, CRL-4001). AGS cell line was cultured in RPMI-1640 medium supplemented with 10% FBS, while BJ-5ta cell line was cultured in high glucose DMEM medium supplemented with 10% FBS. The cells were cultured in an incubator (ESCO, Singapore) at 37 °C supplied with 5% CO_2_ atmosphere. The culture medium was renewed every 2–3 days and cells were passaged when the confluence reached 70–80%.

### Cytotoxicity assay

Firstly, cells were detached from culture surface by 0.25% trypsin- 0.53 mM EDTA, then centrifuged to obtain cell pellet. The pellet was resuspended in fresh medium and cell density was identified using Neubauer chamber (Hirschmann, Germany). After that, 100 μl of cell suspension (10^5^ cells/mL) was transferred into each well of 96-well plate and incubated overnight. Next, the medium was replaced with fresh medium containing extracts at experimental concentrations. In initial screening step, the extract concentration of 40 μg/ml was used, while in IC_50_ identification step, the concentrations ranged from 0.8 to 100 μg/ml. The final concentration of DMSO in each well was kept less than 0.1% (v/v). Control untreated cells were treated with 0.1% (v/v) DMSO. Blank wells containing medium and extract (or 0.1% DMSO) were also established. The plate was incubated at 37 °C for 48 h, then 5 μl of 5 mg/ml MTT was transferred into wells. After 4-h incubation, 60 μl of lysis buffer (30% (w/v) SDS, 0.03 N HCl) and 90 μl of DMSO (99.9% (v/v)) were added. The absorbances were measured at wavelength of 550 nm. The percentage growth inhibition was calculated following the formula:

$$\mathrm{I}\%=100\%-\left({\mathrm{OD}}_{\mathrm{treated}}-{\mathrm{OD}}_{\mathrm{blank}}\right)/\left({\mathrm{OD}}_{\mathrm{control}}-{\mathrm{OD}}_{\mathrm{blank}}\right)\ \mathrm{x}\ 100\%$$

The whole assay was independently repeated in triplicate. IC_50_ (concentration of 50% growth inhibition) values were interpolated by GraphPad Prism 6. Meanwhile, selectivity index (SI) value was calculated by the ratio of IC_50_ of extract on normal cell line to IC_50_ of extract on cancer cell line.

### Analysis of cell growth

Cells were seeded into wells at density of 10^5^ cells/mL and treated with extract at IC_50_ concentration. The cell growth of treated and untreated samples was indirectly quantified through OD values of MTT assay at different time points of 0 h, 24 h, 48 h, and 72 h.

### Analysis of cell morphology

Cells were seeded into wells at density of 10^5^ cells/mL and treated with extract at IC_50_ concentration. Cell morphologies of treated and untreated samples were captured by inverted microscope Nikon Eclipse TiU (Nikon, Japan) after 48 h treatment.

### DAPI staining and immunofluorescence staining with anti-cleaved caspase 3 antibodies

After being seeded into wells at density of 10^5^ cells/mL, cells were treated with extract at IC_50_ concentration. After 48-h incubation, cells were fixed in 4% (v/v) paraformaldehyde for 10 mins, permeabilized with 0.1% (v/v) Triton-X100 for 5 mins.

For DAPI-dye staining, cells were incubated with DAPI solution (1:250 (v/v)) for 15 mins, then washed thrice with PBS solution before being visualized under a fluorescence microscope (Nikon, Japan).

For immunofluorescence staining, after the permeabilization, experimental wells were blocked by 10% (v/v) NGS for 30 mins. Subsequently, cells were incubated with anti-cleaved caspase-3 antibodies (1:200 (v/v)) for 1 h, washed thrice with PBS solution before being simultaneously incubated with secondary antibodies (1:500 (v/v)) and DAPI solution (1:250 (v/v)) for 1 h. Finally, the cells were washed with PBS solution and observed under the fluorescence microscope.

### Quantitative RT-PCR

Cells were seeded into wells at density of 10^5^ cells/mL and treated with extract at IC_50_ concentration. After 48-h incubation, total RNA was extracted using TriSure Kit following manufacturer’s instructions. Next, 500 ng of total RNA was converted into cDNA using Tetro cDNA synthesis Kit and oligo-dT primers. Finally, qPCR was conducted by Lightcycler 96 System (Roche Diagnostics, Germany) using SensiFAST HRM Kit and 1 μl of cDNA template. Thermal cycling conditions were as follows: 1 cycle denaturation of 95 °C for 2 mins, 45 cycles of 95 °C for 5 s, annealing temperature for 10s, 72 °C for 10s. The whole assay was independently repeated in triplicate.

Relative mRNA expression levels of treated samples compared to control samples were calculated by the formula 2^-ΔΔCt^. In the current study, genes including *BAX, BAK, BID, APAF-1, BCL-2, TP53* were investigated, accompanied by reference gene *ACTB (β-actin)*. Primer sequences were referred to previous documents [23–27]. Primer sequences were as followed: *BAX-F*: CAAACTGGTGCTCAAGGCCC, *BAX-R*: GGGCGTCCCAAAGTAGGAGA, *BAK-F*: TTTTCCGCAGCTACGTTTTT, *BAK-R*: TGGTGGCAATCTTGGTGAAGT, *BID-F*: TGGACTGTGAGGTCAACAACG, *BID-R*: AGTCTGCAGCTCATCGTAGCC, *APAF1-F*: CACGTTCAAAGGTGGCTGAT, *APAF1-R*: TGGTCAACTGCAAGGACCAT, *BCL2-F*: CTGGTGGACAACATCGCCCT, *BCL2-R*: TCTTCAGAGACAGCCAGGAGAAAT, *TP53-F*: GCCCAACAACACCAGCTCCT, *TP53-R*: CCTGGGCATCCTTGAGTTCC, *ACTB-F*: TCCTGTGGCATCCACGAACT, *ACTB-R*: GAAGCATTTGCGGTGGACGAT.

### Statistical analysis

In this study, experimental data were analyzed by GraphPad Prism 6 software. The results were described as mean ± SD of at least three independent experiments. The non-linear regression curves describing the relationship between logarithm of extract concentrations and I% were established by using GraphPad Prism, from which IC_50_ values were interpolated by the software. The differences of mean were tested by unpaired Student’s t-test (two-tailed). The *p*-value less than 0.05 indicates a statistically significant difference between two groups.

## Results

### Cytotoxicity of EM extracts on AGS cell line

**Fig. 1:**
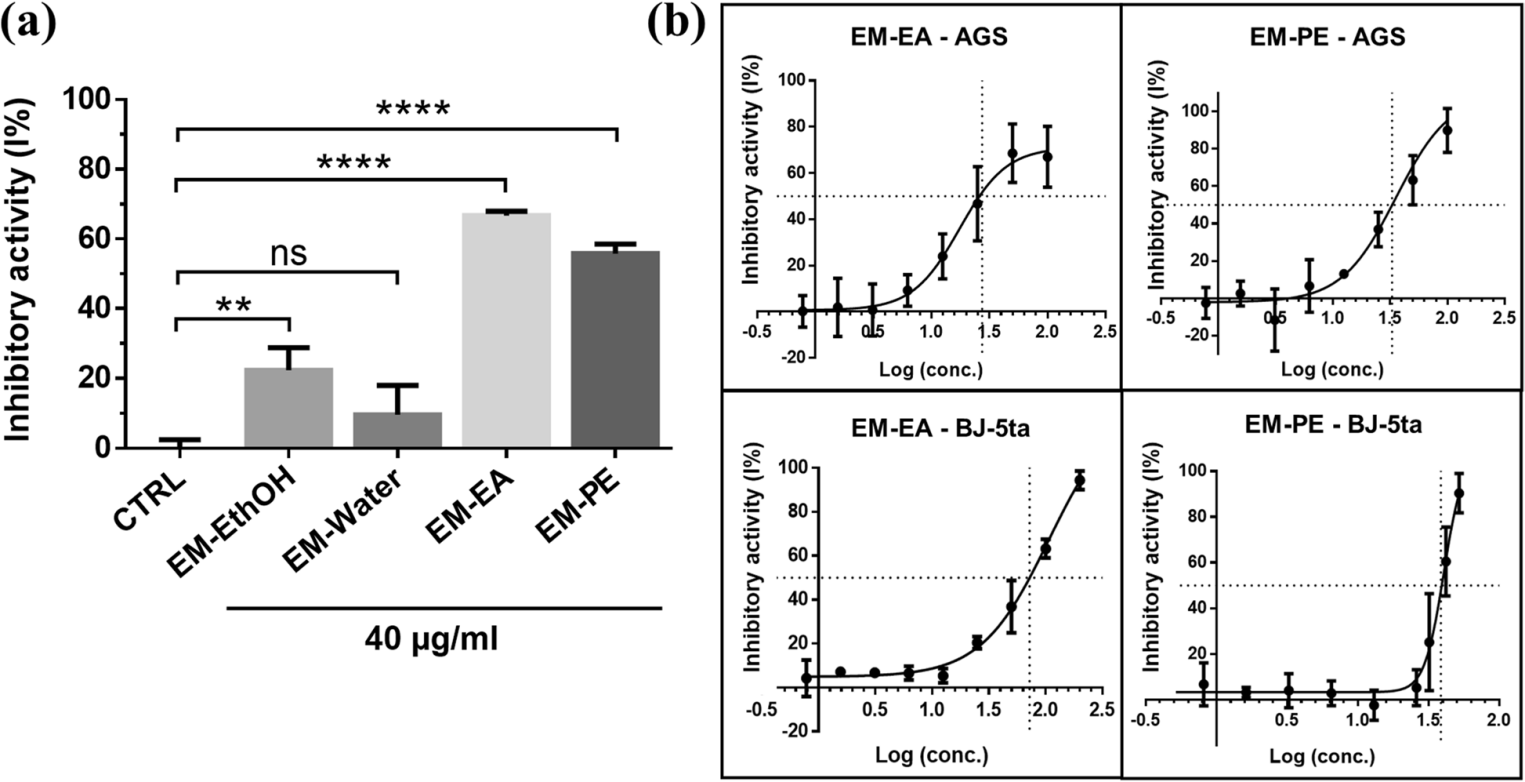
The cytotoxicity of EM extracts. **a** The percentage growth inhibition of EM extracts at concentration of 40 μg/ml on AGS cell line. **b** The percentage growth inhibition of EM-EA and EM-PE extracts at experimental concentrations on AGS and BJ-5ta cell lines. Each value represents mean ± SD of at least three independent experiments. The statistical differences were analyzed by two-tailed unpaired Student’s t-tests (**p < 0.01; ****p < 0.0001; ns, *p* > 0.05)

To investigate the cytotoxicity of EM extracts, the extract concentration of 40 μg/ml was utilized. The results showed that EM-EA and EM-PE extracts exhibited remarkable inhibition (I% = 66.7 and 55.8% respectively) (Fig. 1a). Therefore, EM-EA and EM-PE extracts were subjected to IC_50_ analysis with concentrations ranging from 0.8 to 100 μg/ml.

At the highest concentration (100 μg/ml), EM-EA and EM-PE extract caused 67 and 90% inhibition on AGS cells; meanwhile, for the concentrations less than 10 μg/ml, the extracts exhibited no cytotoxicity (Fig. 1b). The IC_50_ values of EM-EA and EM-PE extracts towards AGS cell line were 27.5 and 37.2 μg/ml, respectively (Table 1). Following the criterion of National Cancer Institute (NCI), a crude extract with IC_50_ value less than 20–30 μg/ml is considered as having highly cytotoxic effect [16, 28]. Interestingly, EM-EA extract was less toxic to BJ-5ta normal fibroblast cell line (IC_50_ = 72.2 μg/ml). The selectivity index (SI) value of extract was used to emphasize the selective effect on cancer cell line compared to normal cell line [29], in which SI ≥ 2 indicates selective toxicity towards cancer cells. The SI values of EM-EA and EM-PE extracts were 2.6 and 1.0 (Table 1). Therefore, EM-EA extract was subjected to further analysis.

**Table 1:** The IC_50_ and the selectivity index values of *Elephantopus mollis* Kunth extracts on AGS cell line

	IC_**50**_ (μg/mL)			Selectivity index (SI)	
	EM-EA	EM-PE	DOX	EM-EA	EM-PE
**AGS**	27.5 ± 10.9	37.2 ± 8.7	0.26 ± 0.02	2.6	1.0
**BJ-5ta**	72.2 ± 12.6	38.5 ± 5.1	1.63 ± 0.63		

The assay was repeated at least three times independently. The SI value of extract was calculated by the ratio of IC_50_ on normal fibroblast cell line (BJ-5ta) to IC_50_ on cancer cell line (AGS). Abbreviations: *DOX* doxorubicin

### Effect of EM-EA extract on cell growth

The AGS cell growth of EM-EA-treated samples was indirectly evaluated through OD_550nm_ values of MTT assay. In this assay, AGS samples were treated with EM-EA extract at IC_50_ concentration, then OD_550nm_ values were measured at time points of 0, 24, 48, and 72 h.

**Fig. 2:**
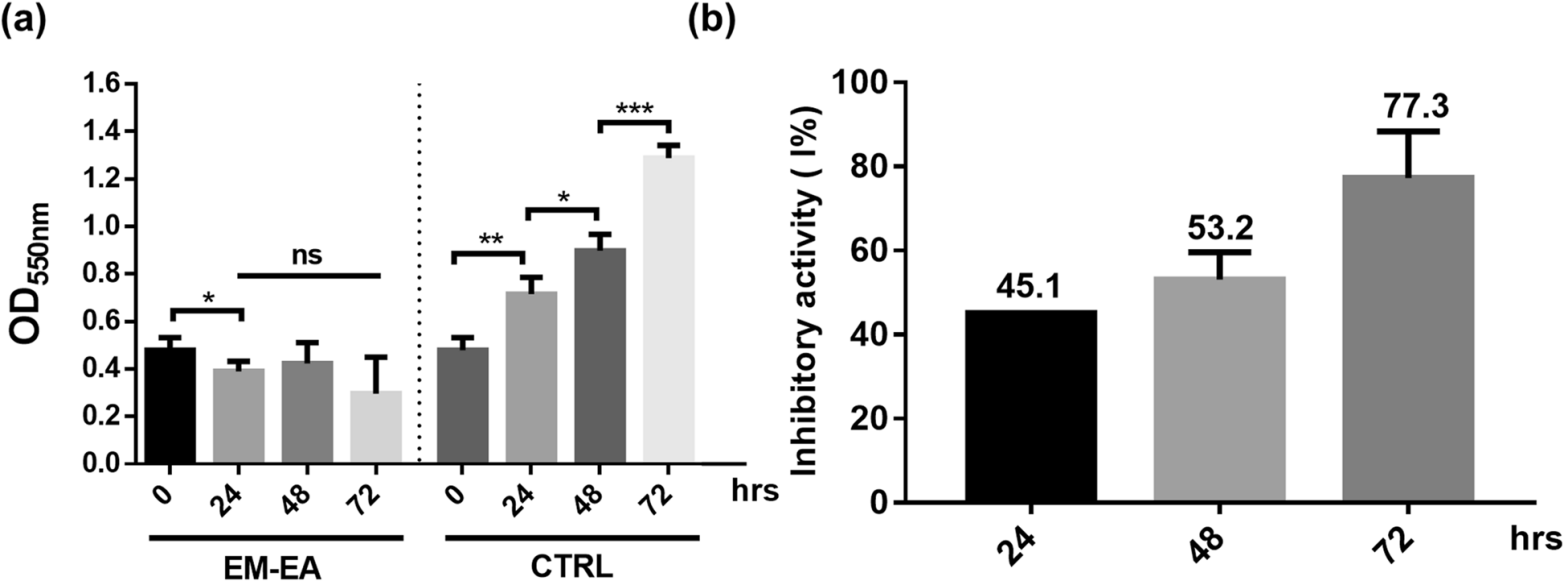
The effects of EM-EA extract on cell growth and percentage growth inhibition of AGS cells during time. **a** The OD_550nm_ values of samples treated with EM-EA extract (27.5 μg/ml) and 0.1% (v/v) DMSO (control) at different time points. **b** The percentage growth inhibition of EM-EA-treated samples when compared with control samples at different time points. Each value represents mean ± SD of four independent experiments. The statistical differences were analyzed by two-tailed unpaired Student’s t-tests (*p < 0.05; **p < 0.01; ***p < 0.001; ns, *p* > 0.05)

The OD_550nm_ values of EM-EA-treated samples decreased by 18.5% at 24 h (*p* = 0.0394) when compared with that at 0 h and remained unchanged from 24 h to 72 h (Fig. 2a). The OD_550nm_ values of untreated samples continuously increased. Particularly, the values at 24 h, 48 h, and 72 h increased 1.5, 1.3, and 1.4 times respectively (*p* = 0.0018, 0.0106, and 0.0001). The inhibitory percentages at 24, 48, and 72 h were 45.1, 53.2, and 77.3% (Fig. 2b). These results suggested that with EM-EA treatment, AGS cells might experience cell deaths or growth inhibition. To elucidate, cell morphology of EM-EA-treated samples was analyzed next.

### Effect of EM-EA extract on cell morphology

**Fig. 3:**
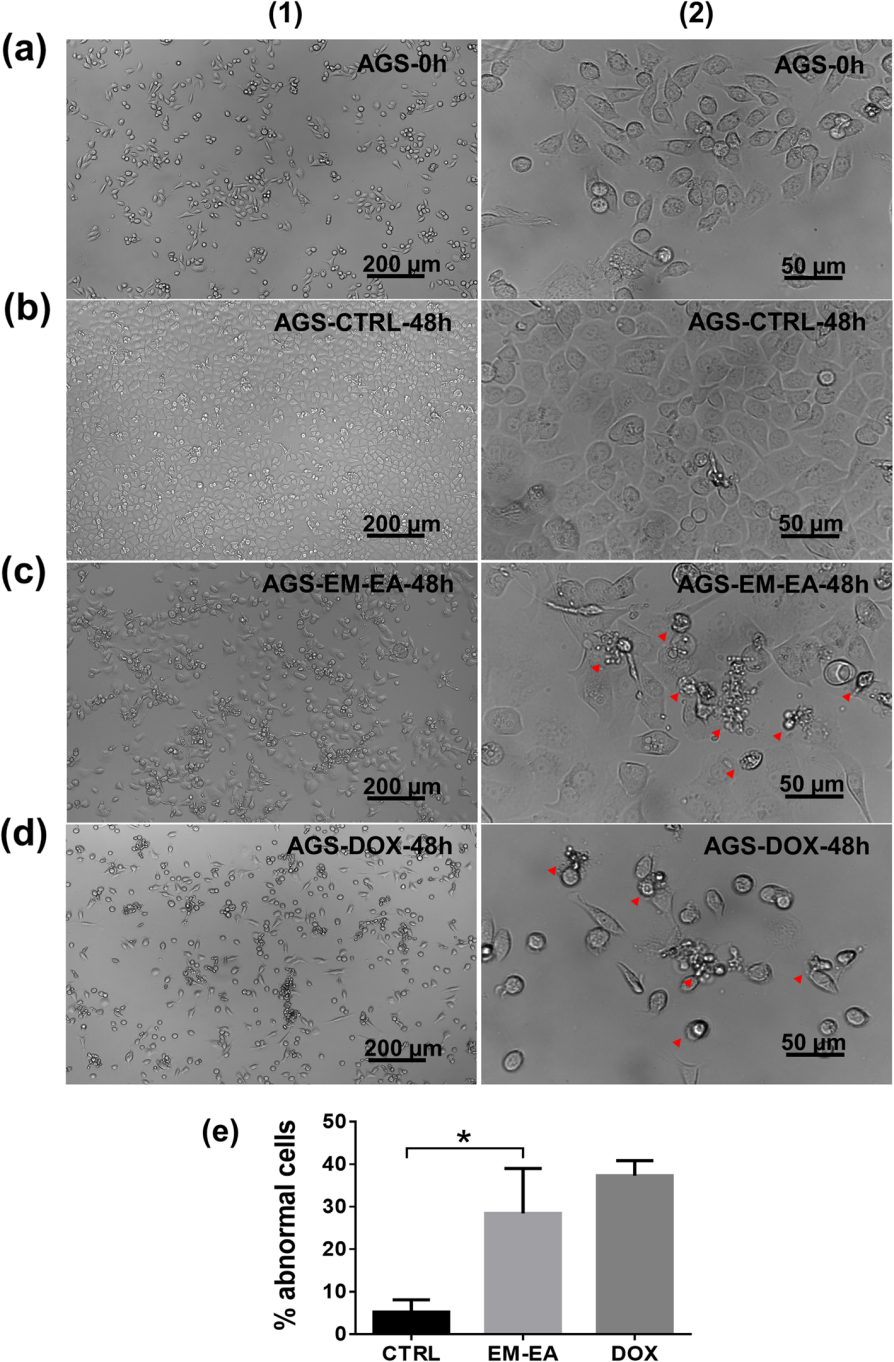
The effect of EM-EA extract on cell morphology of AGS cells. **a** Sample at 0 h before treatment. **b** Control sample treated with 0.1% (v/v) DMSO at 48 h. **c** Sample treated with EM-EA extract (27.5 μg/ml) at 48 h. **d** Sample treated with doxorubicin (0.3 μg/ml) at 48 h. **e** The percentage of abnormal cells in cell population of control samples (*n* = 3), EM-EA-treated samples (*n* = 3), and DOX-treated samples (*n* = 3) at 48 h. Each value represents mean ± SD. The statistical difference was analyzed by two-tailed unpaired Student’s t-tests (*p < 0.05). (1) Images captured at 100x magnification. (2) Images captured at 400x magnification. Arrowheads point cells with abberant morphological features

The changes of cell morphology were observed under an inverted microscope. As illustrated in Fig. 3b, for control samples, cells spread on culture surface with characteristic morphology. Meanwhile, at IC_50_ concentration of EM-EA extract, treated cells turned round, shrunk and be surrounded by several small bodies (Fig. 3c). These features are similar to cell morphology of apoptotic death depicted previously [30]. The percentage of cells owning abnormal morphology of EM-EA-treated samples was 28.4%, which is 5.5 times as high as that of untreated samples (5.2%) (*p* = 0.0215) (Fig. 3e).

Besides, in this study, doxorubicin, a chemotherapeutic agent and apoptosis inducer [31], was also used for comparison. The results showed that EM-EA-treated samples got the same morphological features as doxorubicin-treated ones (Fig. 3d). For that reason, it is suggested that cells treated with EM-EA extract might experience apoptotic deaths.

### Nuclear fragmentation with EM-EA treatment

It is well-known that apoptotic cells have nuclear fragmentation [32]. Thus, to examine, EM-EA-treated samples were stained with DAPI DNA-binding dye, then visualized under fluorescence microscope.

**Fig. 4:**
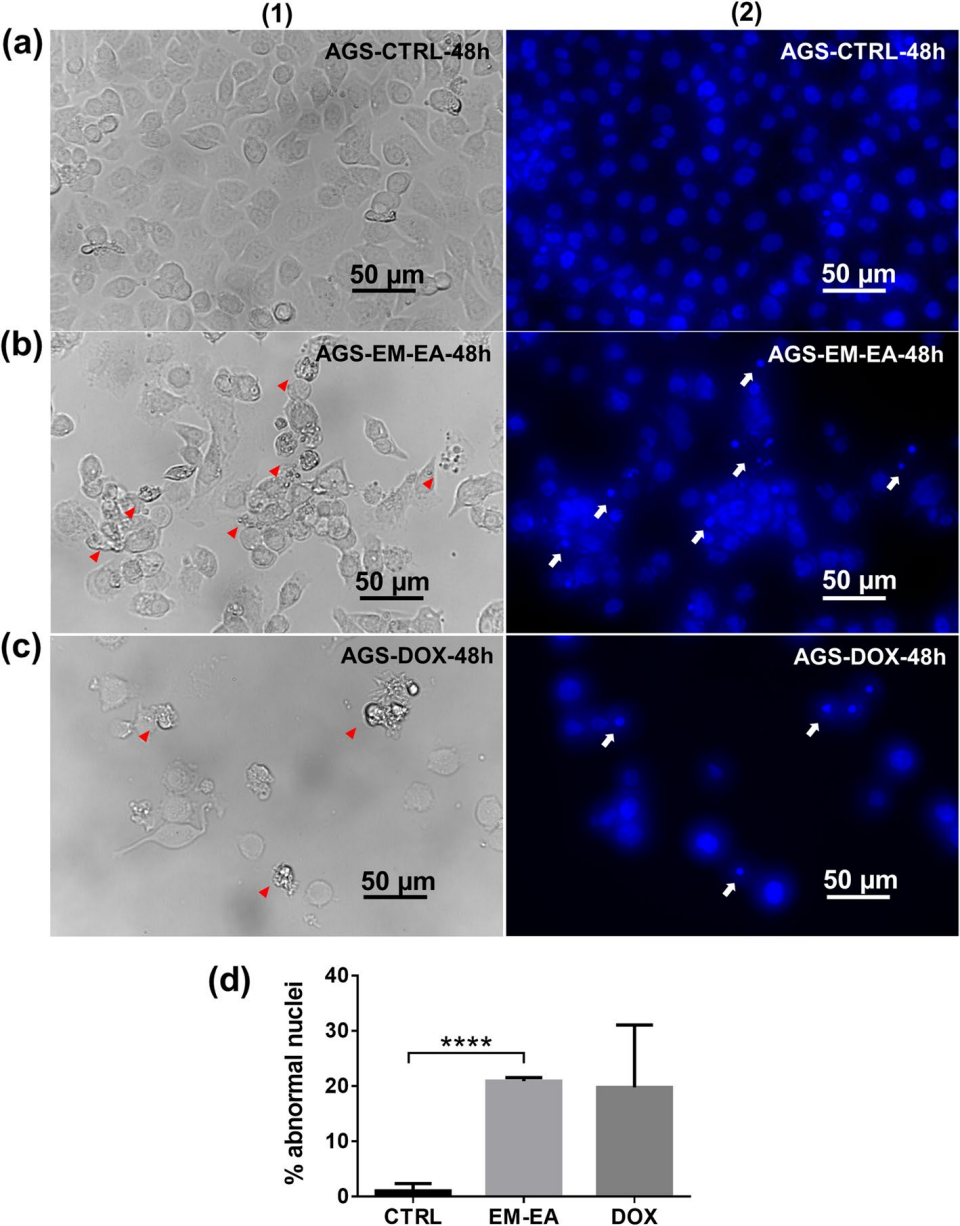
The nuclear morphology of AGS cells treated with EM-EA extract. **a** Control sample treated with 0.1% (v/v) DMSO at 48 h. **b** Sample treated with EM-EA extract (27.5 μg/ml) at 48 h. **c** Sample treated with doxorubicin (0.3 μg/ml) at 48 h. **d** The percentage of abnormal nuclei of control samples (*n* = 3), EM-EA-treated samples (*n* = 3), and DOX-treated samples (*n* = 2) at 48 h. Each value represents mean ± SD. The statistical difference was analyzed by two-tailed unpaired Student’s t-tests (****p < 0.0001). (1) Bright-field images. (2) Fluorescence-field images. Arrowheads and arrows point cells with abberant cell morphology and nuclear fragmentation respectively

As shown in Fig. 4b, the nuclei of EM-EA-treated samples had specific phenomena such as shrinkage, condensation, and fragmentation at locations of abnormal cells; meanwhile, for control samples, cell nuclei were fairly identical in shape and size (Fig. 4a). Besides, the nuclear shrinkage and condensation were also observed in doxorubicin-treated samples (Fig. 4c). The percentage of abnormal nuclei of EM-EA-treated samples was 20.9%, which is greater than that of untreated ones (1.1%) (*p* < 0.0001) (Fig. 4d). These results strongly support that EM-EA extract activated apoptotic deaths in AGS cells.

### Caspase-3 activation induced by EM-EA extract

**Fig. 5:**
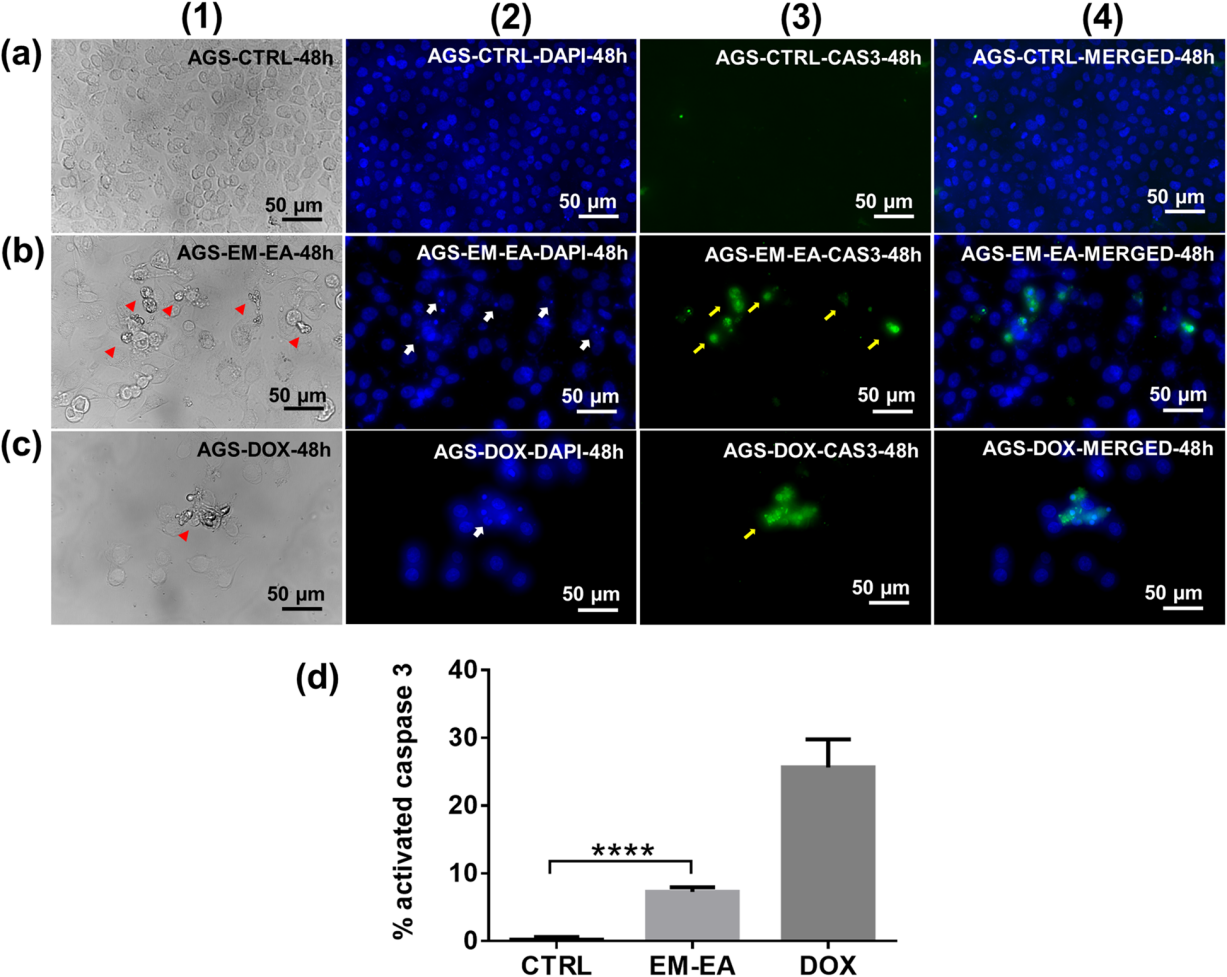
The caspase-3 activation of AGS cells treated with EM-EA extract. **a** AGS control cells treated with 0.1% (v/v) DMSO at 48 h. **b** AGS cells treated with EM-EA extract (27.5 μg/ml) at 48 h. **c** AGS cells treated with doxorubicin (0.3 μg/ml) at 48 h. **d** The percentage of activated-caspase 3-expressing cells of control samples (*n* = 3), EM-EA-treated samples (*n* = 3), and DOX-treated samples (*n* = 2) at 48 h. Each value represents mean ± SD. The statistical difference was analyzed by two-tailed unpaired Student’s t-tests (****p < 0.0001). Columns (1), (2), (3), (4) represent images of cell morphology (bright field), nuclear morphology (blue), activated-caspase-3 (green), and merging of (2) and (3) (blue and green). Arrowheads, arrows, and thin arrows point abberant cell morphology, nuclear fragmentation, and caspase-3 activation respectively

Caspase-3, a cysteine-aspartic acid protease, is an executive enzyme which can trigger apoptotic cell death by activating other enzymes to disrupt nuclear structure, cause cell shrinkage and induce membrane blebbing. The activation of caspase-3 is considered as marker of apoptosis [33]. In our study, activated-caspase-3 signals appeared at same places with fragmented nuclei of both EM-EA-treated and doxorubicin-treated samples (Fig. 5b, c). In comparison with control untreated cells, no signal of caspase-3 activation was observed (Fig. 5a). The percentage of activated-caspase-3-expressing cells of EM-EA-treated samples was 7.3%, while that of untreated samples was 0.2% (Fig. 5d). Thus, the results demonstrate that EM-EA extract triggered apoptosis in AGS cells through caspase-3 activation.

### The mechanism of apoptosis activation by EM-EA extract in AGS gastric cancel cells

Apoptosis can be triggered by intrinsic signaling pathway through mitochondria and/or extrinsic pathway through membrane receptors [34]. In this study, to elucidate the mechanism of apoptotic activation, the expression of pro-apoptotic (*BAX, BAK, BID, APAF-1*) and anti-apoptotic genes (*BCL-2*) was investigated by qRT-PCR.

**Fig. 6:**
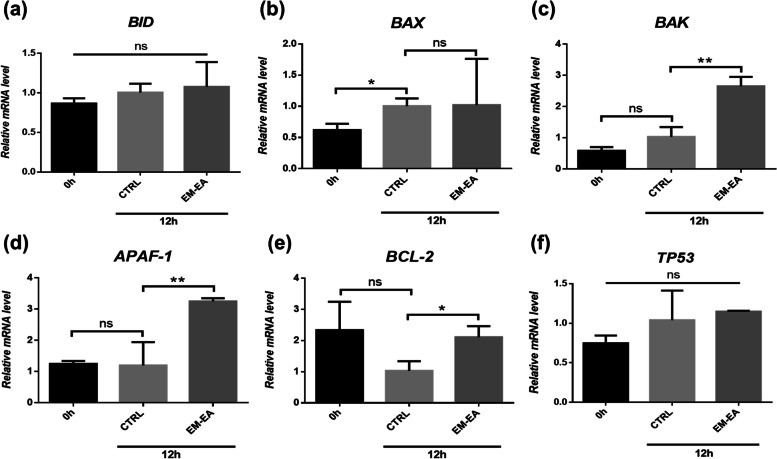
The mRNA levels of apoptosis-related genes of AGS cells treated with EM-EA extract (27.5 μg/ml). **a-f** The mRNA levels of *BID*, *BAX*, *BAK*, *APAF-1*, *BCL-2*, *TP53* genes at 12 h. Each value represents mean ± SD of three independent experiments. The statistical differences were analyzed by two-tailed unpaired Student’s t-tests (*p < 0.05; **p < 0.01; ns, *p* > 0.05)

As depicted in Fig. 6a, b, the expression levels of *BID* and *BAX* showed no difference between treated and untreated cells. Notably, *BAK* and *APAF-1* expression levels of treated samples increased 2.57 and 2.71 times (*p* = 0.0029 and 0.009 respectively) when compared with those of untreated ones (Fig. 6c, d). Surprisingly, the mRNA level of *BCL-2* anti-apoptotic gene was also upregulated 2.05 times (*p* = 0.0162) (Fig. 6e), suggesting that the treated cells had response against extract impact. In addition, we also examined the expression of *TP53* gene, encoding a transcriptional factor which can take part in apoptosis through regulating pro-apoptotic genes such as *BAX, BAK, APAF-1* [27, 35, 36]. However, as shown in Fig. 6f, there was no difference in *TP53* expression levels between treated and untreated samples.

In brief, EM-EA extract might trigger apoptosis through the upregulation of *BAK* and *APAF-1* levels in AGS cells.

## Discussion

Although anticancer activity of EM has been studied on liver carcinoma, lung carcinoma, leukemia cell lines, etc., no report on EM activity against gastric cancer has been published so far. Here, the cytotoxic effect of EM-EA extract on AGS gastric cancer cell line was firstly verified with IC_50_ of 27.5 μg/ml, which meets the criterion of highly cytotoxic crude extract (IC_50_ < 20–30 μg/ml) [16, 28]. Importantly, EM-EA extract was less toxic to BJ-5ta, a normal human fibroblast cell line (IC_50_ = 72.2 μg/ml). For chemotherapeutic treatments, beside the efficacy, side effects on normal tissues are also problems to be considered. Thus, an agent causing both selective and high toxicity on cancer cells compared with normal cells would be an ideal candidate for drug development [37]. In the current study, EM-EA extract might be a potential target for further investigation.

Following the extract effects on cell growth, cell morphology, and nuclear morphology helped to evaluate the anticancer mechanism of EM-EA extract on AGS cells. The EM-EA-treated cells experienced apoptotic deaths as evidenced by characteristic features such as cell shrinkage accompanied by small disintegrated bodies, nuclear fragmentation, and caspase-3 activation. According to previous reports, EM-EA extract could trigger apoptosis towards HepG2 liver carcinoma, A549 lung carcinoma, and HL60 leukemia cells [16, 17]. Therefore, our results add that EM-EA extract could also induce apoptosis in AGS gastric cancer cells.

**Fig. 7:**
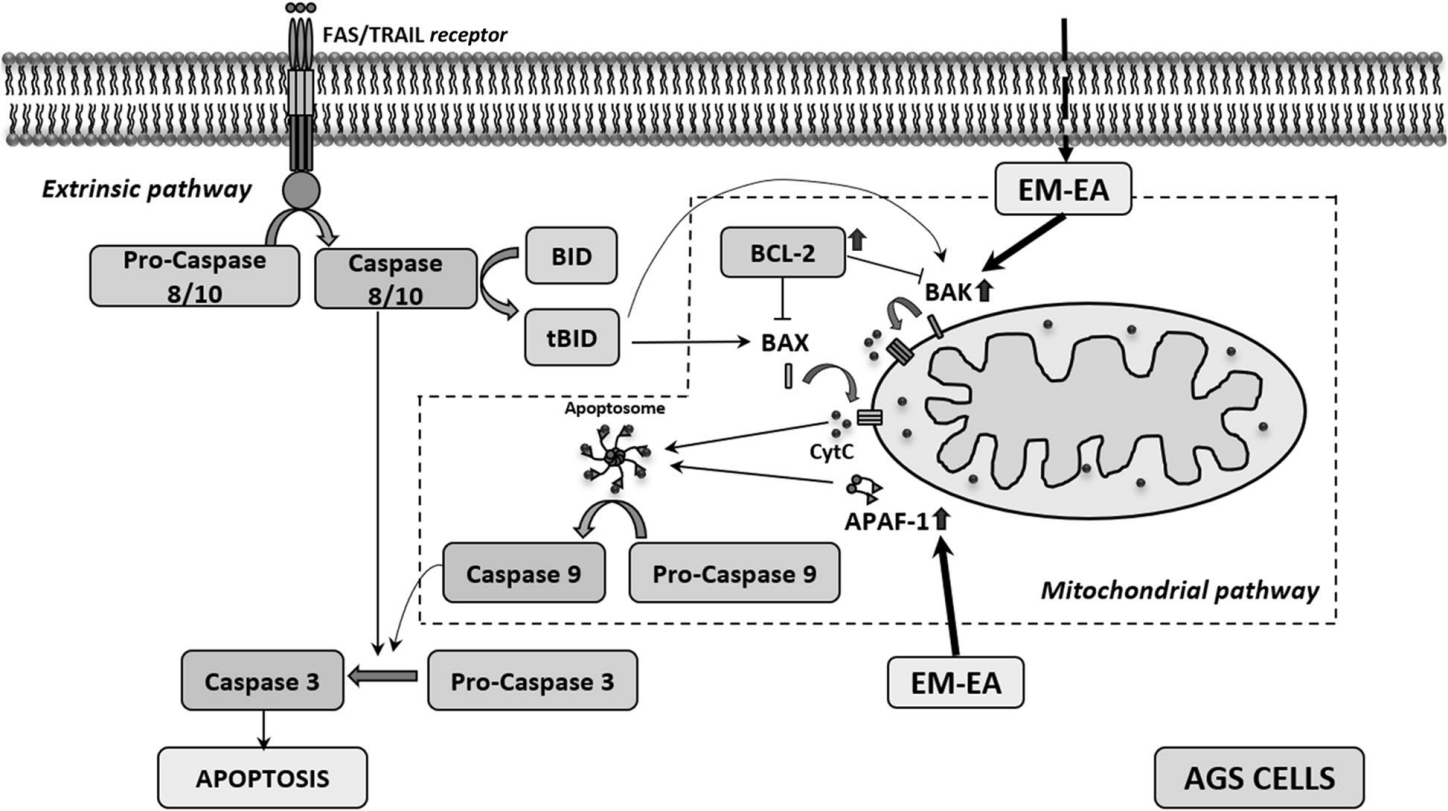
The proposed apoptotic mechanism of AGS gastric cancer cells induced by EM-EA extract

Apoptotic death could be triggered by extrinsic signaling pathway through receptors on cell membrane and/or intrinsic pathway through mitochondria [34]. For extrinsic pathway, once death-inducing ligands such as FasL/TNFa/TRAIL bind to receptors on outer cell membrane, caspase 8/10 is processed, then activate caspase 3 to initiate apoptosis. For intrinsic pathway, the oligomerization of BAX, BAK proteins would be triggered to form pores on mitochondrial outer membrane leading to the release of cytochrome c which then interacts with APAF-1 to form apoptosome for caspase-3 activation. In addition, extrinsic pathway could be connected to intrinsic pathway due to the fact that activated-caspase-8 is able to cleave BID proteins causing BAX/BAK oligomerization. On the contrary, BCL-2, anti-apoptotic protein, binds to BAX/BAK to prevent the oligomerization in order to protect the integrity of mitochondrial membrane [38, 39]. In this study, with EM-EA treatment, expression levels of BAK and APAF-1 increased 2.57 and 2.71 times, while BAX and BID levels remained unchanged. It has been reported that gastric tumor has reduced-BAK levels when compared with normal mucosa, and the BAK overexpression could induce apoptosis in gastric cancer cells [40]. In addition, the increase in BAK expression indicates good chemotherapeutic response in advanced gastric cancer [41]. Therefore, our results, which recorded the upregulation of *BAK* mRNA level after EM-EA-extract treatment, show an optimistic sign for drug screening to treat gastric cancer. Besides, the simultaneous upregulation of *BAK* and *APAF-1* levels also suggests that EM-EA extract might trigger apoptosis through mitochondrial pathway (Fig. 7). The mRNA expression of P53, a transcriptional factor which could regulate *BAK* and *APAF-1* gene expression, was not different between treated and untreated samples. In this case, *BAK* and *APAF-1* genes might be transcriptionally regulated by another factor, or P53 proteins might be regulated by post-translational modifications leading to their stable presence in cells [42] then prompting transcriptional activation of *BAK* and *APAF-1* genes. Thus, next studies should be conducted to investigate further.

Our results also showed that with EM-EA treatment, the anti-apoptotic gene *BCL-2* was upregulated 2.05-folds. As described above, the cell densities of treated samples decreased at 24 h then remained unchanged from 24 h to 72 h (Fig. 2a). Therefore, it might be that beside the population suffering apoptosis, some cells had resistant response towards the extract impact. There has also been a report on the correlation between increased BCL-2 level and the resistance of chemotherapeutic drugs such as 5-fluorouracil, adriamycin, mitomycin C in gastric cancer [43]. Importantly, despite that resistance, EM-EA extract caused a remarkable inhibition (I% = 77.3% at 72 h) and induced apoptosis in AGS cells due to the upregulation of *BAK* and *APAF-1* expression levels.

## Conclusion

Gastric cancer is one of the most common cancers with leading mortality rate. Screening of novel agents from natural sources, including medicinal herbs, is an approach in treatment research. In this study, ethyl acetate extract of *Elephantopus mollis* Kunth species was proved to be a potential candidate against gastric cancer. Specifically, EM-EA extract exhibited high cytotoxicity (IC_50_ = 27.5 μg/ml) and could induce apoptosis towards AGS gastric cancer cell line. The initial investigation of mechanism pointed that apoptosis activation might be triggered through mitochondrial pathway due to the upregulation of pro-apoptotic genes such as *BAK* and *APAF-1*.

## Data Availability

The datasets used and/or analysed during the current study are available from the corresponding author on reasonable request.

## Abbreviations

CAS3: Activated caspase-3
CTRL: Control
DOX: Doxorubicin
EM: *Elephantopus mollis* Kunth
EM-EA: Ethyl acetate extract of *Elephantopus mollis* Kunth
EM-EtOH: Ethanol extract of *Elephantopus mollis* Kunth
EM-PE: Petroleum ether extract of *Elephantopus mollis* Kunth
EM-Water: Aqueous extract of *Elephantopus mollis* Kunth
I%: The percentage growth inhibition
IC_50_: 50% inhibitory concentration
OD_550nm_: Optical density at wavelength 550 nm
SI: Selectivity index
